# Communicating distress: suicide threats/gestures among clinical and community youth

**DOI:** 10.1007/s00787-022-01960-5

**Published:** 2022-02-28

**Authors:** Kealagh Robinson, Christian Scharinger, Rebecca C. Brown, Paul L. Plener

**Affiliations:** 1 School of Psychology, Victoria University of Wellington, Wellington, New Zealand; 2 Department of Child and Adolescent Psychiatry, Medical University of Vienna, Vienna, Austria; 3 Department of Child- and Adolescent Psychiatry and Psychotherapy, University of Ulm, Ulm, Germany

**Keywords:** Suicide gesture, Suicide threat, Self-harm, Suicidal behavior, Non-suicidal self-injury, Adolescence

## Abstract

Although self-injurious thoughts and behaviors are a global health concern, little is known about suicidal threat/gesture(s) where a person leads others to believe they want to end their lives when they have no intention to do so. This study assessed the lifetime prevalence of self-injurious thoughts and behaviors among both community adolescents (*n* = 1117) and in clinical youth (*n* = 191). Suicide threats/gestures were common among youth; 12.2% of community adolescents and 18.0% of clinical youth reporting having made a suicide threat/gesture, most commonly in the context of other self-injurious thoughts and behaviors. Across both samples, suicide threats/gestures were not uniquely associated with suicide attempts, and youth who reported suicide threats/gestures in the context of a history of self-harm or suicide plan(s) were no more likely to report a history of suicide attempt(s). Suicide threats/gestures were distinguished from suicide attempts in that they primarily fulfilled positive social functions, rather than autonomic functions. Findings suggest that suicidal threats/gestures are common in both community and clinical youth, and are not uniquely associated with suicide attempts, but rather function to communicate distress to others.

**Supplementary Information:**

The online version contains supplementary material available at 10.1007/s00787-022-01960-5.

Self-injurious thoughts and behaviors (SITBs) are a global public health concern. Globally, nearly 800,000 people die by suicide each year [33], with recent evidence to suggest rates of self-injury among adolescents have increased in recent years [10]. Although the nomenclature of SITBs is the subject of continuing debate [15], SITBs have been distinguished by intent to die into the two superordinate clusters: Suicidal phenomena and non-suicidal phenomena [17]. Suicidal phenomena are further distinguished into suicidal ideation, suicide plans, and suicide attempts, with a suicide attempt being defined as engaging in self-injurious behavior with at least some intent to die [21]. Non-suicidal phenomena are further distinguished into non-suicidal thoughts, non-suicidal self-injury (NSSI), and Suicide Threats/Gestures.^1^ Despite a rich literature investigating the nature of suicidal ideation, suicide plans, suicide attempts, and NSSI [6, 8, 29], suicide threats/gestures—in which an individual verbally or behaviourally leads others to believe they want to end their lives when they have no intention to do so—remains largely unexplored.

Initial investigation has focused on estimating the prevalence of suicide threats/gestures among youth. Within clinical settings, 9.4% of Spanish adolescents receiving outpatient care [3] and 18.9% of German adolescents receiving inpatient care reported a lifetime history of suicide threat/gesture(s) [4]. Similarly, 22.3% of a US youth sample recruited from community and outpatient settings reported a lifetime history of suicide threats/gestures, 12.8% a past year history, and 2.1% a past month history [22]. Focusing on youth recruited from community settings, 1.9% of the National Comorbidity Survey (a nationally representative sample of US participants aged 15–54 years) [23] and 1.8% of a university student sample [32] reported a lifetime history of suicide threats/gestures. Among a diverse sample of US adolescents aged 14–16, 1.5–6.9% reported having made a suicide threat/gesture in the previous 6 months at two time points in a longitudinal study [13]. Thus, as with other SITBs, suicide threats/gestures appear to be more common among youth in clinical settings compared to community settings. Given the initial prevalence estimates of suicide threats/gestures and the importance of adolescence as a key developmental period for the onset and course of SITBs generally [28, 30], a more thorough understanding of suicide gestures/threats that moves beyond prevalence rates to focus on the clinical characteristics and underlying motives is critical.

Currently, the demographic features of young people who report a suicide threat/gesture history are poorly understood. Although gender differences in SITBs generally are common [1, 26], preliminary evidence for gender differences in suicide threats/gestures is mixed. One study found that women were more likely to report a lifetime history of suicide threats/gestures than were men [23], another found no difference [25], and yet another found that young women were less likely to report suicide threats/gestures than men [13]. To date, no research has assessed suicide threats/gestures among gender diverse individuals. Compared to participants who had made a suicide attempt, participants with a history of suicide threats/gestures were less likely to report psychiatric symptoms, comorbidity of clinical diagnoses, or a history of multiple physical and sexual assault, suggesting that suicide threats/gestures may be associated with less psychopathology [23]. In a similar manner, among a sample of German inpatient adolescents, participants’ mental state was rated as having the least influence on suicide threats/gestures compared to all other forms of SITBs [4].

Moreover, the relationship between suicide threats/gestures and other SITBs remains unclear. College students who had engaged in NSSI were more likely to also report suicide gestures/threats [32], and adults with a borderline personality disorder diagnosis who reported a suicide gesture/threat were 3.02 times more likely to also report a suicide attempt [31]. In contrast, among secondary school adolescents, neither suicidal ideation, suicide attempts, nor NSSI predicted subsequent suicidal threats/gestures, and suicidal threats/gestures themselves did not predict subsequent suicide ideation or suicide attempts [13]. A growing body of research has investigated the functions which may maintain suicide threats/gestures. The four-function model proposes that self-injurious behavior can be maintained by four distinct reinforcement processes: automatic negative reinforcement (i.e., to reduce aversive internal states), automatic positive reinforcement (i.e., to generate positive emotions or stimulation), social negative reinforcement (i.e., to escape from uncomfortable social situations), and social positive reinforcement (i.e., to gain support from others) [24]. When it comes to the functions of SITBs, the previous research has found suicidal ideation, suicide plans, suicide attempts, and NSSI serve largely automatic negative and automatic positive functions [3, 4, 9, 22]. In contrast, preliminary evidence suggests that suicide threats/gestures are largely characterized by social positive functions [9].

Given the lack of consensus on suicidal threats/gestures and the links to suicidal attempts and NSSI, understanding the behavior is critical not only to improve understanding of SITBs in general, but also to inform clinical decision-making in how to respond therapeutically to the behavior. In the current study, we assessed the prevalence and characteristics of suicide threats/gestures among youth recruited from community and clinical settings, before testing the relationships between suicide threats/gestures with other SITBs, focusing in particular on the relationship between suicidal threats/gestures and suicide attempt(s).

## Method

### Participants

The community adolescent sample comprised 1117 adolescents recruited from high schools in two studies (Sample A *n* = 665, Sample B *n* = 452). As a composite community sample, 52.7% of participants identified as female and 47.3% identified as male, and the average age was 14.83 (SD = 0.63). The clinical youth sample comprised 191 adolescents and young adults recruited from an adolescent inpatient clinic (*n* = 139) following psychiatric hospitalization for self-injury (*n* = 52). As a composite clinical sample, participants tended to be older adolescents (*M* age = 17.08, SD = 3.36), and identify as female (75.4% identified as female, and 24.6% identified as male). Demographic information for each sample and comparisons for the prevalence of SITBs for each sample is provided in the Supplementary Materials.

### Measures

*Self-injurious thoughts and behaviors* were assessed across the clinical and community samples using two different measures. The community sample reported their lifetime SITB history using the German version of the Self-Harm Behavior Questionnaire (SHBQ) [5, 14]. The SHBQ is a self-report measure that assesses the lifetime prevalence of NSSI (‘Have you ever hurt yourself on purpose? e.g., scratched yourself with fingernails or a sharp object’), suicidal ideation (‘Have you ever talked or thought about committing suicide?’), suicide plan (‘Did you have a specific plan(s) for how you would try to kill yourself?’), suicide attempts (‘Have you ever attempted suicide?’), and suicide threats (‘Have you ever threatened to commit suicide?’). Follow-up questions assess further details about the SITBs, such as the frequency and method(s).

The clinical sample reported their lifetime SITB history on the German version of the Self-Injurious Thoughts and Behaviors Interview (SITBI-G) [4, 22]. The SITBI-G is a structured interview that assesses the lifetime prevalence of NSSI (‘Have you ever intentionally harmed yourself without wanting to die, for example, cutting or burning?’), suicidal ideation (‘Have you ever had thoughts of killing yourself?’), suicide plan (‘Have you ever actually made a plan to kill yourself?’), suicide attempts (‘Have you ever made an actual attempt to kill yourself in which you had at least some intent to die?’), and suicide gestures (‘Have you ever done something to lead someone to believe that you wanted to kill yourself when you really had no intention of doing so?’). Follow-up questions assess further details about the behaviors, such as the frequency and method(s). Participants who reported a history of suicide threats/gestures and suicide attempts reported the extent these behaviors served four classes of functions; automatic positive reinforcement (‘How much did you make this attempt(s) to feel something, because you were feeling numb or empty?’), automatic negative reinforcement (‘How much did you make this attempt(s) as a way to get rid of bad feelings?’), social positive reinforcement (‘How much did you do this to communicate with someone else or to get attention?’), and social negative reinforcement (‘How much did you do this to get out of doing something or to get away from others?’). Participants are invited to respond on a 5-point scale ranging from ‘0—very little’ to ‘4—very strongly’.

Although the SHBQ assesses verbal suicide threats and the SITBI assesses behavioral suicide gestures, in line with theoretical conceptualizations [21], we refer to these interchangeably as ‘suicide threats/gestures’. It is also worth noting that among the clinical youth, who reported a history of suicide gestures, when asked to describe the gesture several provided descriptions of verbal threats (e.g., ‘saying ‘I’m going to kill myself” in an argument’, and ‘sending a text message containing a suicide threat’), suggesting that participants did not distinguish between behavioral gestures and verbal threats. Across both community and clinical samples, a presence of a lifetime history for each item was coded as a binary variable (0 ‘no history’ or 1 ‘lifetime history’). For youth who reported a history of suicide threats/gestures, the specificity of the suicide threat/gesture was coded as a binary variable (0 ‘unspecified threat/gesture’ or 1 ‘specific threat/gesture’).

### Procedure

The community adolescent sample was comprised of data collected from two German high school samples [27, 34] and the clinical youth sample was comprised of an inpatient clinical sample [11] and a follow-up study of youth who were hospitalized for self-injury [12]. All studies were carried out in accordance with the Declaration of Helsinki and were approved by the institutional review boards of the University of Ulm. Further ethical approval, recruitment processes, and data collection procedure details are provided elsewhere [11, 12, 27, 34].

### Analytic procedures

Across both samples, we first calculated the prevalence of each type of self-injurious thoughts or behaviors and compared prevalence groups by age and gender. Second, we calculated the comorbidity of other self-injurious thoughts or behaviors among participants who made a suicide threat/gesture. Next, we conducted correlational analysis among all self-injurious thoughts or behaviors across both the community and clinical samples using non-parametric Kendall’s Tau to account for binary variables with small sample sizes. We then used hierarchical logistic regression models to test whether suicidal threats/gestures moderate the relationship between other SITBs and suicide attempts. For the clinical sample only, we compared participants suicide threats/gestures and suicide attempts by method and function (these data were not available for the community sample). Finally, an ANOVA was conducted to test for differences in suicide threats/gestures and suicide attempt(s) functions among clinical youth. Across all statistical analyses, alpha was set at 0.05 with < 0.07 considered a statistical trend. Materials, de-identified data, and analysis code are available for review: https://osf.io/5w6vh/?view_only=c407686ec5c946b683b70d97af08912f.

## Results

### Prevalence of suicidal threats/gestures

Focusing first on the community adolescent sample, SITBs were common among school-based youth; 23.6% of participants reported a history of NSSI, 32.4% suicidal ideation, 12.5% suicide plan(s), 5.6% suicide attempt(s), and 12.2% reported having made suicide threat/gesture(s). Table 1 presents age and gender differences in lifetime prevalence of SITBs across both community and clinical samples. Compared to those with no lifetime history, participants who reported a lifetime history of NSSI, suicidal ideation, suicide attempt(s), and suicide threat/gesture(s) were older and more likely to be young women. Of the community adolescents who reported a suicide threat/gesture history, the majority (68.1%) reported doing so 1–2 times, 22.2% 3–4 times, and 9.6% reported doing so 4 or more times. The method of suicide communicated by suicide threats/gestures was highly variable, 41.7% involved direct self-injury behavior, 14.6% ingesting substances, 12.6% a severe accident, and 31.1% an unspecified fatal act. Participants who reported a lifetime history of suicide threat/gesture(s) also typically reported a lifetime history of other SITBs; 61.4% had a history of NSSI, 84.1% suicidal ideation, 48.1% suicide plan(s), and 20.0% had a history of suicide attempt(s). Notably, only 9.8% participants who reported a history of suicide threat/gesture(s) reported no other self-injurious thoughts or behaviors.

**Table 1:** Age and gender differences in lifetime prevalence of self-injurious thoughts and behaviors across community and clinical samples

	Community sample				Clinical sample			
	Lifetime history *M* (SD)	No history *M* (SD)	Hedges’ *g*	*p* Value	Lifetime history *M* (SD)	No history *M* (SD)	Hedges’ *g*	*p* Value
Age								
NSSI	14.94 (0.70)	14.79 (0.61)	0.24	0.001	17.83 (3.64)	15.45 (1.81)	0.74	< 0.001
Suicidal ideation	14.88 (0.63)	14.79 (0.62)	0.14	0.035	17.33 (3.45)	15.81 (2.56)	0.46	0.021
Suicide plan	14.90 (0.67)	14.80 (0.62)	0.16	0.096	17.74 (3.69)	16.42 (2.89)	0.40	0.007
Suicide attempt	15.10 (0.75)	14.81 (0.62)	0.46	< 0.001	17.88 (3.71)	16.56 (3.02)	0.40	0.010
Suicide threat/gesture	14.94 (0.67)	14.81 (0.63)	0.21	0.026	16.91 (3.45)	17.10 (3.37)	0.05	0.766

	Community sample				Clinical sample			
	% Women with lifetime history	% Men with lifetime history	Cramer’s *V*	p Value	% Women with lifetime history (%)	% Men with lifetime history	Cramer’s *V*	*p* value
Gender								
NSSI	30.9	15.5	0.18	< 0.001	79.2	36.2	0.40	< 0.001
Suicidal ideation	40.3	23.3	0.18	< 0.001	87.5	72.3	0.18	0.014
Suicide plan	14.4	10.4	0.06	0.055	52.4	34.0	0.16	0.028
Suicide attempt	7.1	3.9	0.07	0.021	47.2	17.0	0.27	< 0.001
Suicide threat/gesture	15.9	8.0	0.12	< 0.001	17.6	19.1	0.02	0.811

*NSSI* non-suicidal self-injury

Next, we focus on the prevalence of SITBs among youth recruited from clinical settings. Reflecting greater psychopathology among clinical youth relative to community adolescents, 68.6% of clinical youth reported a history of NSSI, 83.8% suicidal ideation, 47.9% suicide plan(s), 39.8% suicide attempt(s), and 18.0% reported a history of suicide threat/gesture(s). As for the community adolescent sample, clinical youth who reported a lifetime history of NSSI, suicidal ideation, suicide plans, and suicide were older and more likely to be women than participants without a history (see Table 1). In contrast, clinical youth who reported a lifetime history of suicide threats/gestures did not differ by age or gender compared to those with no lifetime history. Again, the method of suicide communicated by suicide threats/gestures was highly variable: 23.5% reported threatening to cut with a sharp object, 17.6% jumping from a height, 8.8% with medication, and 50.0% reported making an unspecified threat. As among community adolescents, clinical youth who reported a history of suicide gesture(s) also typically reported a lifetime history of other SITBs; 67.6% reported a history of NSSI, 82.4% suicidal ideation, 42.4% suicide plan(s), and 38.2% a history of suicide attempt(s). Only 8.8% of clinical youth who reported a suicide threat/gestures(s) history did so in the absence of other self-injurious thoughts or behaviors.

### Relationship between suicide threats/gestures and other self-injurious thoughts and behaviors

Given the high comorbidity between suicide threats/gestures and other SITBs across both community and clinical samples, we now consider how suicide threats/gestures relate to other SITBs. Table 2 displays non-parametric associations among SITBs, split by sample. Among community adolescents, all forms of SITBs—including suicide threats/gestures—showed moderate positive correlations with one another. In contrast, among clinical youth although NSSI, suicidal ideation, suicide plans, and suicide attempts all showed strong, positive correlations with one another, suicide threats/gestures were unrelated to any other SITBs.

**Table 2:** Nonparametric correlations among self-injurious thoughts and behaviors across community and clinical samples

	NSSI	Suicide ideation	Suicide plan	Suicide attempt
Suicide ideation				
Community sample	0.38*	–	–	–
Clinical sample	0.47*	–	–	–
Suicide plan				
Community sample	0.31*	0.55*	–	–
Clinical sample	0.40*	0.37*	–	–
Suicide attempt				
Community sample	0.31*	0.23*	0.35*	–
Clinical sample	0.34*	0.36*	0.47*	–
Suicide threat/gestures				
Community sample	0.33*	0.43*	0.42*	0.23*
Clinical sample	– 0.01	– 0.02	– 0.05	– 0.02

Clinical sample *n* = 191, community sample *n* = 1089

*NSSI* non-suicidal self-injury

*p < 0.001

Focusing on youth with a suicide threat/gesture history, we next assessed whether the nature of the suicide threat/gesture was associated with other SITBs. Community adolescents who reported making a specific suicide threat/gesture (compared to an unspecified threat/gesture) were more likely to report a history of NSSI (*r*_τ_ = 0.52, *p* < 0.001), with a statistical trend to suggest that they are also more likely to report a history of suicide attempt(s) (*r*_τ_ = 0.24, *p* = 0.076). All community adolescents who reported a history of suicide gestures/threats also reported a history of suicide ideation and suicide plans. In contrast, clinical youth who reported making a specific suicide threat/gesture (compared to a unspecified threat/gesture) were no more likely to report a history of NSSI (*r*_τ_ = 0.11, *p* = 0.527), suicidal ideation (*r*_τ_ = 0.14, *p* = 0.419), suicide plans (*r*_τ_ = − 0.07, *p* = 0.693), or suicide attempts (*r*_τ_ = − 0.04, *p* = 0.832). Of the 13 clinical youth who reported methods for both their suicide gesture and suicide attempt,^2^ the majority (76.9%, *n* = 10) reported different methods for their suicide gesture(s) and suicide attempt(s), suggesting that making a specific threat/gesture likely is not associated with suicide attempt methods.

Although NSSI, suicidal ideation, and suicide plans are all behaviors of significant clinical concern, suicide attempts are the behavior with the greatest clinical risk given their high risk of lethality. Next, we tested whether a lifetime history of suicide threats/gestures was *uniquely* associated with suicide attempts for both community adolescents and clinical youth. We conducted an exploratory binomial logistic regression in which age, gender, NSSI, suicidal ideation, suicide plans, and suicide threats/gestures were entered into a logistic regression as predictors of lifetime suicide attempt(s). Table 3 displays the regression results. Among community adolescents, a history of suicide threat/gesture(s) or suicide ideation were unrelated to suicide attempt(s), whereas NSSI and suicide plan(s) were positively associated with suicide attempt(s). Among clinical youth, all participants who reported suicide attempt(s) also reported suicidal ideation and so suicidal ideation was excluded from the model. As for community adolescents, suicide plan(s) were uniquely associated with a history of suicide attempt(s) among clinical youth, with a statistical trend to suggest that NSSI history may also be uniquely associated with suicide attempts. Again, suicide threat/gesture(s) was unrelated to suicide attempts among clinical youth. Taken together, this pattern of results suggests that positive association between suicide threats/gestures and suicide attempts among community adolescents can be accounted for by their shared variance with NSSI and suicide plans.

**Table 3:** Regression analysis predicting suicide attempts by self-injurious thoughts and behaviors across community and clinical samples

	Community sample		Clinical sample	
	OR (95% CI)	*p*	OR (95% CI)	*p*
	*χ*^2^(6, *n* = 1000) = 125.59, *p* < 0.001, Nagelkerke *R*^*2*^ = 0.34		*χ*^2^(5, *n* = 188) = 58.14, *p* < 0.001, Nagelkerke *R*^2^ = 0.36	
Age	1.43 (0.95, 2.28)	0.083	1.04 (0.93, 1.15)	0.487
Gender	1.21 (0.62, 2.35)	0.582	2.98 (1.13, 7.83)	0.027
Suicide threat/gesture	1.48 (0.74, 2.98)	0.272	1.07 (0.43, 2.70)	0.881
NSSI	6.70 (3.21, 14.00)	< .001	2.26 (0.90, 5.68)	0.083
Suicidal ideation	1.20 (0.48, 3.00)	0.701	–	–
Suicide plan	5.69 (2.57, 12.58)	< 0.001	6.08 (2.95, 12.52)	< 0.001

Gender is coded where male = 0, female = 1

*NSSI* non-suicidal self-injury

Next, we considered whether a suicide threat/gesture(s) history that occurs in the context of other lifetime SITBs is associated with a history of suicide attempt(s). Given that NSSI and suicide plan(s) were uniquely associated with suicide attempt(s), we assessed whether suicide threats/gestures moderated the relationship of these behaviors with suicide attempts. For both the community and clinical samples, we conducted two exploratory binomial logistic regression models in which age, gender, suicide threat/gesture(s), NSSI, and suicide plan(s) were entered as predictors of suicide attempt(s) in the first step of the model, followed by the interaction term between suicide threat/gesture(s) and NSSI (Model 1) and suicide plan(s) (Model 2) entered at step two. The interaction between suicide threat/gesture(s) and NSSI failed to explain additional variance in suicide attempts in either the community (*χ*^2^(1, *n* = 1000) = 1.47, *p* = 0.225) or clinical sample (*χ*^2^(1, *n* = 188) = 1.09, *p* = 0.296). In a similar manner, the interaction between suicide threat/gesture(s) and suicide plans failed to explain additional variance in suicide attempts in either the community (*χ*^2^(1, *n* = 1000) = 2.44, *p* = 0.118) or clinical sample (*χ*^2^(1, *n* = 188) = 1.69, *p* = 0.193). Taken together, no evidence was found to suggest that suicidal threat/gesture(s) that occur alongside a history of NSSI or suicide plans explain addition variance in suicide attempt(s) among either community or clinical youth.

### Functions of suicide threats/gestures

**Fig. 1:**
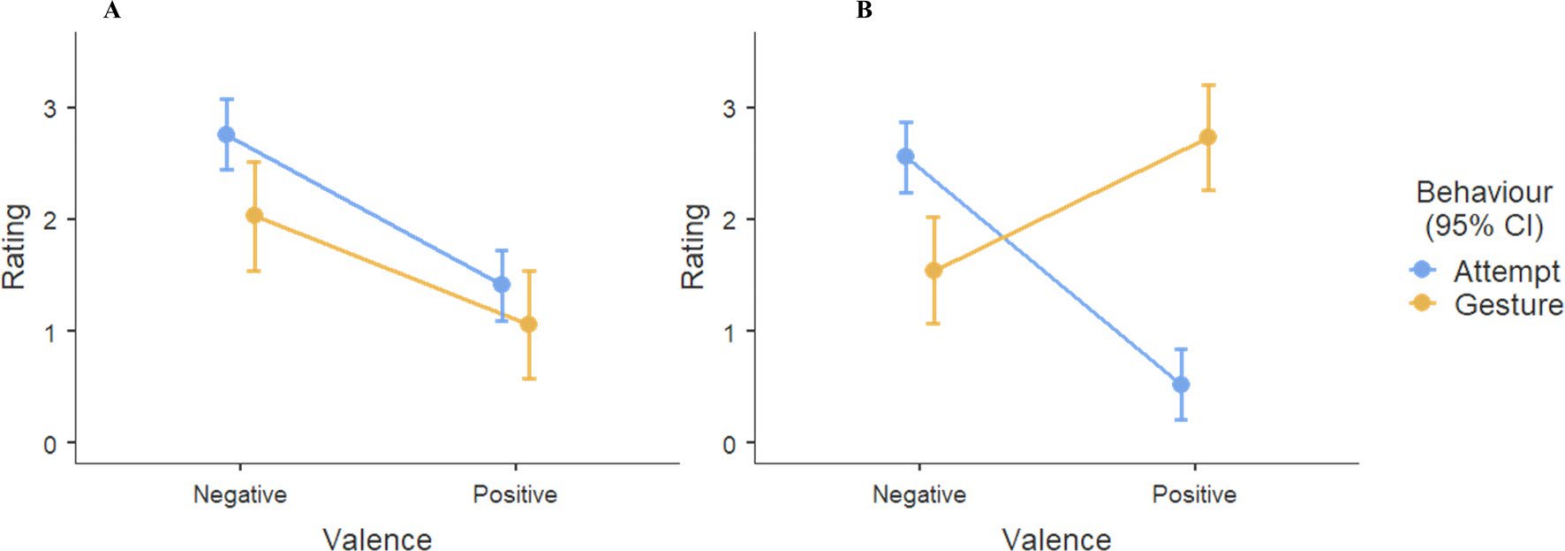
Ratings of automatic functions (**A**) and social functions (**B**) by valence, for suicide attempts and suicide threats/gestures among clinical youth. Suicide attempts *n* = 34, suicide threat/gestures *n* = 76

Finally, we focus on the functions of suicide threats/gestures compared to suicide attempts among clinical youth. We conducted a mixed-effect regression with age and gender as between-subjects predictors, and function (automatic, social), valence (positive, negative), and behavior as within-subject predictors. In addition to these fixed effects, we also included a random intercept for participants to account for the 13 participants who provided data for both suicide threats/gestures and attempts (and thus, these measures are not independent). A three-way interaction was found between function, valence, and behavior, *t*(335.62) = 5.26, *p* < 0.001. To investigate this interaction further, we split by Function to conduct two mixed-effect regressions with valence (positive, negative) and behavior (suicide threat/gesture, suicide attempt) fixed effects, and a random intercept for participants (see Fig. 1).

For automatic functions, clinical youth rated negative automatic functions (*M* = 2.03, SD = 1.49) higher than positive automatic functions (*M* = 1.06, SD = 1.37), *F*(1, 116.93) = 43.53, *p* < 0.001. We found no difference in autonomic functions by behavior (*F*(1, 199.06) = 3.48, *p* = 0.064), or any interaction between Behavior and Valence (*F*(1, 116.93) = 1.14, *p* = 0.287). For social functions, the valence (*F*(1, 126.17) = 4.97, *p* = 0.028) and behavior (*F*(1, 183.71) = 8.12, *p* = 0.005) main effects were qualified by an crossover interaction between valence and behavior (*F*(1, 124.54) = 71.00, *p* < 0.001). Critically, suicide threats/gestures served *fewer* negative social functions and considerably *greater* positive social functions than did suicide attempts (negative social functions: suicide threat/gestures *M* = 1.56, SD = 1.37, suicide attempts *M* = 2.56, SD = 1.53, *t*(64.70) = 3.31, *p* = 0.002, Hedges *g* = 0.67; positive social functions: suicide threat/gestures *M* = 2.74, SD = 1.62, suicide attempts *M* = 0.53, SD = 0.99, *t*(44.31) = 7.36, *p* < 0.001, Hedges’ *g* = 1.80). This pattern of results suggests that, although both suicide threats/gestures and suicide attempts function in a similar manner to up- and down-regulate internal states, socially suicidal threats/gestures function to gain desirable responses from others, while suicide attempts function to escape interpersonal demands.

## Discussion

Despite growing understanding of the nature of NSSI, suicidal ideation, suicide plans, and suicide attempts [6, 8, 29], to date the nature of suicide threats/gestures remains largely unexplored. This study assessed the prevalence of suicide threats/gestures among youth recruited from community and clinical settings, before investigating the association between suicide threats/gestures and other forms of SITBs, and (in the clinical sample only) how the functions of suicide threats/gestures compare to those of suicide attempts. Finally, we assessed whether a history of threats/gestures, either alone or in the context of a history of NSSI or suicide plans, were uniquely associated with a suicide attempt history.

A lifetime history of suicide threat/gestures was common within both community adolescent (12.2%) and clinical youth samples (18.0%). These findings extend the previous studies which reported 1.9% lifetime prevalence among a nationally representative sample of 15–54 years old [19] and between 9.4 and 22.3% lifetime prevalence among inpatient and outpatient adolescent samples [3, 4, 7, 22]. As with other forms of SITBs, community adolescents who reported making a suicidal threat/gesture were older and more likely to be women than those who had not, suggesting a gender effect that has been demonstrated previously [23]. In comparison, no age or gender differences were found among the adolescents and young adults in the clinical sample.

Suicide threats/gestures were highly comorbid with other forms of SITBs; 90.2% of community adolescents with a history of suicide threat/gesture(s) and 91.2% of the clinical sample also reported a lifetime history of one or more other forms of self-injurious thoughts or behaviors. This high comorbidity raises questions for how previous research has operationalised suicide threats/gestures. Two previous studies investigating suicide threats/gestures classified participants into disparate groups (e.g., either the suicide attempt group, or the suicide threat/gesture group) based on either the most recent SITB episode [23] or the most clinically serious behavior [32]. Given that in the current study, fewer than 10% of participants who reported making a suicide threat/gesture had done so in the absence of other forms of self-injurious thoughts or behaviors, this classification is likely to underestimate the true prevalence and nature of suicide threats/gestures.

Although previous research has highlighted that a history of NSSI increases the risk of suicidal thoughts and behaviors [29, 30], the association between suicidal threats/gestures was poorly understood. Notably, the pattern of associations between suicide threats/gestures and other SITBs was found to differ across samples in this study. Among community adolescents, suicide threats/gestures were positively associated with all other SITBs. In contrast, among clinical youth suicide threats/gestures were unrelated to all other SITBs, perhaps reflecting the greater prevalence of SITBs in this sample (and thus, a restricted range). Critically, across both community adolescent and clinical youth sample, after accounting for other SITBs, suicide threats/gestures were unrelated to suicide attempts. In addition, suicide threat/gesture in the context of a history of either NSSI or suicide plans were unrelated to lifetime suicide attempt(s) in either sample. These findings align with the understanding of SITBs as put forward by Nock [21], in which behaviors with suicidal intent (i.e., suicidal ideation, suicide attempts, and suicide plans) are delineated from behaviors without suicidal intent (i.e., suicide threats/gestures, NSSI, and NSSI thoughts). Given that suicidal intention is inherent in the definition of suicide attempts used in the current study, these findings suggest that suicide threats/gestures may not reflect an explicit suicidal process per se, but rather may serve as marker of distress more generally. This is especially interesting given the continuing debate on the links between different self-injurious behaviors. There has been a long discussion about the connections between different self-injurious behaviors. Kreitmann et al. [18] noted more than 50 years ago that “it appears that what is required is a term for an event in which the patient simulates or mimics suicide […]. Yet the ‘attempted suicide’ patient is not usually addressing himself to the task of self-destruction” (p. 747) and suggested this behavior be described as ‘parasuicide’. Kreitmann et al., also emphasized that these behaviors could serve a communication function [19]. Overtime, the term “parasuicide” comes to describe all forms of intentional, non-fatal self-injury (i.e., both suicide attempts and acts without suicide intent) [20]. This umbrella nomenclature is similar to both the concept of “deliberate self-harm”, which refers to self-damaging acts such as self-injury or self-poisoning, regardless of the motive or suicidal intent and the broader concept of SITBs [21]. In our work, we assessed the relationships between more specific subgroups of SITBs (e.g., suicide attempts and threats/gestures) to better understand of the connections between them. Given that NSSI, which predominantly serves an emotion regulation function for the majority of youth who self-injure, has been identified as predictor of subsequent suicidal behavior [2], focusing on functions of the respective behaviors can inform the discussion about the interplay between these behaviors.

Among the clinical sample in our study, the psychological function of suicide threats/gestures diverged from that of suicide attempts. Consistent with previous research [3, 4, 9, 22], youth reported that their suicidal threats/gestures fulfilled primarily positive social functions (i.e., to gain desirable responses from others), while suicide attempts fulfilled negative social functions (i.e., escape from interpersonal demands). Together, findings provide support to the classification of suicidal threats/gestures as ‘non-suicidal’ as opposed to suicidal thoughts and behaviors [16].

### Limitations and future research directions

Taken together, our findings indicate that suicidal threats/gestures are not uniquely associated with suicide attempts, but instead co-occur, perhaps reflecting elevated psychological distress in general. However, this conclusion comes with two key caveats. First, the community adolescent and clinical youth samples differed by age and assessed lifetime SITBs using different measures, limiting direct comparison between the two. These measurement differences may explain why we found positive correlations between suicide threats/gestures and other SITBs among community adolescents, but no relationship between suicide threats/gestures and other SITBs among clinical youth. Therefore, rather than reflecting a difference by sample type, it may well be that suicide *threats* are associated with other SITBs, but suicide *gestures* are not. Second, relying on cross-sectional measures means that we are unable to investigate the temporal relationships between suicidal threats/gestures and other SITBs.

Given that suicide threats/gestures require a person to whom the threat/gesture is communicated, subsequent research should focus on describing the interpersonal context in which a suicide threat/gesture is made, and to whom. Qualitative research exploring how people respond to suicide threats/gestures, both immediately following the behavior and more long term, would be critical in understanding the impact of suicide threats/gestures within interpersonal relationships. Future research should also consider whether established risk factors for suicide attempts also predict subsequent suicide threats/gestures.

### Clinical implications

Despite these caveats, this study has two key implications for clinical care. First, the prevalence of suicide threats/gestures among both the community adolescents and clinical youth demonstrates that suicide threats/gestures are common among youth and should be routinely screened for. Given the overlap in presentations between lifetime suicide threats/gestures and other SITBs, clients who report having made a suicide threat/gesture should also receive suicide risk assessment as a clinical standard. Clinical management for youth who present with a history of suicide gestures should differ by suicidal intent. Patients who make suicide threats/gestures while also experiencing high suicidal intent need to be provided with a high level of (inpatient) care and surveillance. In contrast, youth who make suicide threats/gestures *without* also experiencing acute suicidal intent, might best profit from other modes of care, such as outpatient treatment and safety planning. This underscores that the self-defined intent of the patient is leading clinical decision-making.

Second, the greater endorsement of positive social functions of suicide threats/ gestures suggests that working to improve a client’s capacity to communicate their needs may be valuable in reducing suicide threats/behaviors. This certainly holds true also for suicide attempts, as the fostering of communication skills if often used in successful therapeutic approaches toward reducing suicidality. Therapeutic strategies like Dialectical Behavioral Therapy for Adolescents (DBT-A) or family-centered therapeutic interventions have demonstrated efficacy in reducing self-injury and suicidal ideation [16, 17] and thus may be reasonable interventions to establish alternative skills to replace suicidal threats/gestures. Since suicidal threats/gestures appear to be more interpersonally directed and to serve social functions rather than interpersonal functions, clinical management in emergency situations should address this aspect and therapeutic interventions should possibly include peers and family members.

### Conclusions

Importantly, given that a substantial proportion of adolescents and young adults report having made a suicide threat/gesture, more empirical investigations are needed to better understand the nature of this behavior and to better inform optimal clinical and community responses when a young person makes suicide threat/gesture(s). Given the high overlap between suicide threats/gestures with NSSI, suicidal ideation, suicide plans, and suicide attempts, a history of suicide gestures/threats should be taken as a clinical signal to warrant further suicide assessment.

## Code, data, and materials

https://osf.io/5w6vh/?view_only=c407686ec5c946b683b70d97af08912f

## Supplementary Information

Below is the link to the electronic supplementary material.

Supplementary file1 (DOCX 26 KB)

## Footnotes

1 Although ‘suicide threats/ gestures’ is the most common and precise term in recent suicidology research [2, 3, 6, 12, 14, 19]. We use the term here pragmatically to maintain consistency with the literature.

2 Participants reported engaging in the following suicide attempt methods; 50.7% (*n* = 38) medication overdose, 32.0% (*n* = 24) cutting, 12.0% (*n* = 9) jumping from a high place, 9.3% (*n* = 7) poisoning, 5.3% (*n* = 4) train or car accident, 2.7% (*n* = 2) suffocation, and 1.3% (*n* = 1) hanging.
