# Supplementary material for: Communicating distress: suicide threats/gestures among clinical and community youth
Source: Eur Child Adolesc Psychiatry. 2022 Feb 28;32(8):1497–506. doi: 10.1007/s00787-022-01960-5 (PMC10326133; doi:10.1007/s00787-022-01960-5)
Supplement: Supplementary file 1 — Supplementary file1 (DOCX 26 KB) [file 787_2022_1960_MOESM1_ESM.docx]

# **SUPLEMENTARY MATERIALS**

**Communicating distress: Suicide threats/gestures among clinical and community youth**

Kealagh Robinson^1^*, Christian Scharinger^2^*, Rebecca C. Brown ^3, 4^ & Paul L. Plener^2, 3^†

1 School of Psychology, Victoria University of Wellington, Wellington, New Zealand

2 Department of Child and Adolescent Psychiatry, Medical University of Vienna, Vienna, Austria

3 Department of Child- and Adolescent Psychiatry and Psychotherapy, Medical University of Ulm, Ulm, Germany

4 Private practice, Ludwigsburg, Germany

* These authors contributed equally

† Corresponding author: Univ.-Prof. Dr. Paul L. Plener, MHBA, Department of Child and Adolescent Psychiatry, Medical University of Vienna, 01 40400 30110, [paul.plener@meduniwien.ac.at](mailto:paul.plener@meduniwien.ac.at)

## **Comparisons between community samples**

The two high school samples did not differ in age (Sample A: *M* age = 14.85, *SD* = 0.58; Sample B: *M* age = 14.81, *SD* = 0.66), *t*(1042.96) = 0.88, *p =* .377, Hedges’ *g* = 0.05, equal variances not assumed), although there was a greater proportion of men in Sample A than in Sample B (53.8% compared to 42.9%, χ^2^(1, *n* = 1117) = 12.84, *p* < .001, Cramer’s *V*= 0.11). Both samples reported similar proportions of participants who had engaged in non-suicidal self-injury (20.7% compared to 25.6%, χ^2^(1, *n* = 1109) = 3.46, *p* = .063, Cramer’s *V*= 0.06) and who had made a suicide attempt (4.2% compared to 6.5%, χ^2^(1, *n* = 1092) = 2.54, *p* = .111, Cramer’s *V*= 0.05). However, compared to Sample A, a greater proportion of Sample B reported having experienced suicidal ideation (36.4% compared to 25.5%, χ^2^(1, *n* = 1044) = 13.27, *p* <.001, Cramer’s *V*= 0.11), made a suicide plan (15.8% compared to 7.0%, χ^2^(1, *n* = 1031) = 17.34, *p* <.001, Cramer’s *V*= 0.13), and having made a suicide threat (15.7% compared to 6.7%, χ^2^(1, *n* = 1081) = 19.70, *p* <.001, Cramer’s *V*= 0.14).

**Comparisons between clinical samples**

The follow-up sample was significantly older (*M* age = 21.50, *SD* = 2.61, adolescent clinic *M* age = 15.43, *SD* = 1.72, *t*(68.35) = 15.55, *p* < .001, Hedges’ *g* = 3.03, equal variances not assumed) and had a greater proportion of women (94.2% compared to 68.3%, χ^2^ (1, *n* = 191) = 13.67, *p* < .001, Cramer’s *V*= 0.27), than the adolescent clinic sample. In addition, the follow-up sample included a greater proportion of participants who a lifetime history of NSSI (100% compared to 60.3%, χ^2^ (1, *n* = 191) = 32.73, *p* < .001, Cramer’s *V*= 0.41), suicidal ideation (96.2% compared to 79.1%, χ^2^ (1, *n* = 191) = 8.06, *p* = .005, Cramer’s *V*= 0.21), suicide plans (62.7% compared to 42.4%, χ^2^ (1, *n* = 190) = 6.16, *p* = .013, Cramer’s *V*= 0.18), and suicide attempts (53.8% compared to 34.5%, χ^2^ (1, *n* = 191) = 5.89, *p* = .015, Cramer’s *V*= 0.18). However, samples did not differ by the proportion of participants who reported having made a suicide gesture (16.0% compared to 18.7%, χ^2^ (1, *n* = 189) = 0.18, *p* =.669, Cramer’s *V*= 0.03).
